# Quantitatively integrating molecular structure and bioactivity profile evidence into drug-target relationship analysis

**DOI:** 10.1186/1471-2105-13-75

**Published:** 2012-05-04

**Authors:** Tianlei Xu, Ruixin Zhu, Qi Liu, Zhiwei Cao

**Affiliations:** 1 Department of Bioinformatics, Tongji University, 200092, Shanghai, China

## Abstract

**Background:**

Public resources of chemical compound are in a rapid growth both in quantity and the types of data-representation. To comprehensively understand the relationship between the intrinsic features of chemical compounds and protein targets is an essential task to evaluate potential protein-binding function for virtual drug screening. In previous studies, correlations were proposed between bioactivity profiles and target networks, especially when chemical structures were similar. With the lack of effective quantitative methods to uncover such correlation, it is demanding and necessary for us to integrate the information from multiple data sources to produce an comprehensive assessment of the similarity between small molecules, as well as quantitatively uncover the relationship between compounds and their targets by such integrated schema.

**Results:**

In this study a multi-view based clustering algorithm was introduced to quantitatively integrate compound similarity from both bioactivity profiles and structural fingerprints. Firstly, a hierarchy clustering was performed with the fused similarity on 37 compounds curated from PubChem. Compared to clustering in a single view, the overall common target number within fused classes has been improved by using the integrated similarity, which indicated that the present multi-view based clustering is more efficient by successfully identifying clusters with its members sharing more number of common targets. Analysis in certain classes reveals that mutual complement of the two views for compound description helps to discover missing similar compound when only single view was applied. Then, a large-scale drug virtual screen was performed on 1267 compounds curated from Connectivity Map (CMap) dataset based on the fused similarity, which obtained a better ranking result compared to that of single-view. These comprehensive tests indicated that by combining different data representations; an improved assessment of target-specific compound similarity can be achieved.

**Conclusions:**

Our study presented an efficient, extendable and quantitative computational model for integration of different compound representations, and expected to provide new clues to improve the virtual drug screening from various pharmacological properties. Scripts, supplementary materials and data used in this study are publicly available at http://lifecenter.sgst.cn/fusion/.

## Background

To comprehend relationship between intrinsic characteristics of chemical compound and the compound interaction with protein target is an essential task to evaluate potential protein-binding function for virtual drug screening. Similarity relationship between compounds can be characterized differently, depending on different aspects of features to be measured. The similarity measurement of small molecules has been the focus of essentially every compound-based approach to design or identify novel drug candidates [1]. However, in the process of novel drug screening, the representation of a compound varies dramatically, which results in different similarity measurements. Such similarity difference has given rise to distinct candidate compound similarity ranking lists with only generally about 15% overlap [1]. It is demanding and necessary if information from multiple data sources can be integrated together to produce a comprehensive representation and assessment of similarity relationship between small molecules [2], thus expected to boost the results of virtual drug screening.

Generally, the drug candidates are related to specific targets. The investigation on the nature of target-specific structure–activity relationships of molecules should be based on the available data sources concerning structure, activity and target-binding information from a comprehensive and integrative perspective. Fortunately, public resources are in a rapid growth both in the quantity of data and in the type of data-generating, which provide us a great chance to further mine the relationship between compounds and their targets. Besides the classic representations of small molecules, like various fingerprints characterizing compound chemical structure, public high-throughput experimental data representing bioactivity of compounds are boosting with the development of online database, including PubChem (http://pubchem.ncbi.nlm.nih.gov/) [3], Gene Expression Omnibus (GEO, http://www.ncbi.nlm.nih.gov/geo/) [4] and DrugBank (DrugBank, http://drugbank.ca/) [5] etc., which provides an alternative way for molecule characterization based on bioactivity profiles. Several recent studies on the relationship between different compound features claimed that, correlations were proposed between bioactivity profiles and target networks, especially when chemical structures were similar [2,6-8]. By simply combining both public repositories of compound targets and compound bioactivity, these studies indicates that comparison of bioactivity profile can provide insight into the mode of actions (MOA) at the molecular level, which will facilitate the knowledge-based discovery of novel compounds. However although various relationship were found between multiple features, no effective quantitative integrating methods was proposed or evaluated to combine these multi-view features. Inspired by previous works, two important and interesting computational issues are needed to investigate: (1) is there a quantitative relationship between compound features (bioactivity profile and structural feature) and compound target that can be specifically described? (2) Since the former works implicated that an integration of multiple compound features may result in a better measurement of target-specific compound similarity rather than only one specific type was adopted, how such integration can be optimized to quantitatively and automatically combine information from various views of compound representations, i.e., structural features, bioactivity features and other more? Hereby in our study, we refer such multiple features description and integration for compound as a multi-view data representation and learning problem, and we aim at presenting a quantitative relationship between target-specific compound similarity and multi-view representations of compound features in an efficient multi-view learning schema.

It should be noted that the term “multi-view learning” was initially presented from 3D-object recognition by the machine learning and graphic communities [9]. Naturally as implicated by its name, multi-view learning combines models from different aspects of one identical entity to obtain an overall and comprehensive representation for further study. Multi-view learning was classically introduced as co-training, a semi-supervised learning procedure to distinguish webpages using two different types of data [10]. Thereafter the concept of integration of different information sources has been developed for years in the field of information retrieval [11-13]. On the other side, as an unsupervised-learning method, multi-view clustering algorithms can be divided into two categories in general [14]: (1) Fusion of similarity data by deriving a convex combination of similarities from different views to minimize a given penalty error [15,16]. (2) Fusion of clustering decision derived from each view separately [17,18]. In the clustering process, other techniques like canonical correlation analysis (CCA) [19] and matrix factorization [20] were employed to reduce the feature dimension or reconcile clustering groups. These applications of multi-view learning commonly yield better performance than that of single-view learning. In our study, as both the structure and bioactivity information are two distinguished intrinsic features to describe the small molecule, it is natural to investigate the results with the integration of both the chemical space (molecule structure) and genetic space (bioactivity profile) of molecules for a better evaluation of molecular properties and similarity comparison.

In this study, firstly a data set of 37 compounds (in Additional file 1: Table S1) from previous study based on bioactivity profile similarity [6] were adopted. Two similarity matrix characterizing bioactivity profile and structural similarity were calculated. As we would like to investigate the hierarchical structure of similarity among compounds regarding to multiple data sources, rather than only achieve an integrated ranking decision, a similarity fusion method was employed and modified to automatically optimize the weights of the combination of different similarity data. A hierarchy clustering was produced and discussed based on the fused similarity. Then, in order to evaluate the fusion method on the large scale dataset, Connectivity MAP dataset [21] containing 1267 compounds with their gene expression profile and structure fingerprint representation were used to perform drug virtual screen based on similarity searching. The compound-target interaction in these experiments was also analysed and compared quantitatively to demonstrate the benefits introduced by the integration of multiple data representations.

## Materials and methods

### Algorithm workflow

**Figure 1:**
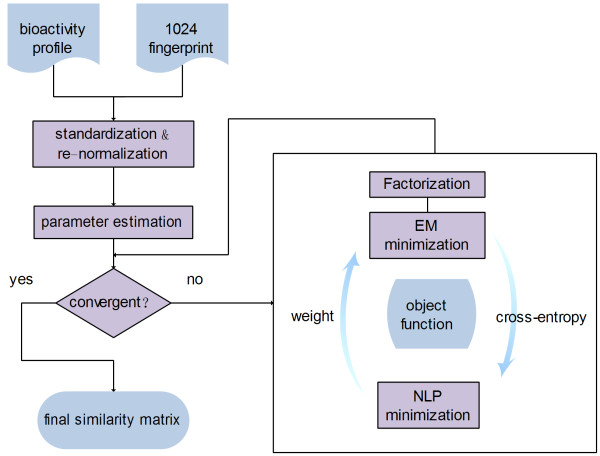
**Workflow of this study.** Initially two similarity matrices of different views were used as input after standardization to the z-value and renormalization. Then a two-step alternative minimization was used to obtain the proper weights for the two similarity matrix in fusion. In the first step, given the initial weights $\alpha =\left(\alpha_{1},\alpha_{2}\right)$. cross-entropy between the input matrices and a combined non-negative factorization was minimized by an EM algorithm. In the second step, given the calculated cross-entropy, the weights were calculated by minimizing the object function, i.e. the cross-entropy and entropy of the weight. The two steps iterate until convergence. The final α was used as an ideal weighing vector that obtains balance between weighted sparseness and informativeness.

The workflow of our analysis is illustrated in Figure 1. The intuition behind this workflow is to automatically identify the weights for two molecule representations in fusion under a mathematical optimization framework. Given two similarity matrix $P_{1}$ and $P_{2}$, weights $\alpha =\left(\alpha_{1},\alpha_{2}\right)$ were to be optimized for a final similarity matrix $p=\alpha_{1}p_{1}+\alpha_{2}p_{2}$. Initially two similarity matrices of different views were used as input after standardization to the z-value and renormalization. Then a two-step alternative minimization was used to obtain the proper weights for the two similarity matrix in fusion. In the first step, given the initial weights $\alpha =\left(\alpha_{1},\alpha_{2}\right)$Cross-entropy between the input matrices and a combined non-negative factorization was minimized by an EM algorithm. In the second step, given the calculated cross-entropy, the weights were calculated by minimizing the object function, i.e. the cross-entropy and entropy of the weight. The two steps iterate until convergence. The final α was used as an ideal weighing vector that obtains balance between weighted sparseness and informativeness. Details are shown below.

### Dataset

#### NCI-60 dataset

In our study, the same data set used in Cheng’s work [6] rather than the up-to-date data is applied for equally comparison purpose, in order to illustrate the superiority of target-relationship analysis with similarity fusion from integration of multi-view information. The NCI-60 data set is available in the PubChem BioAssay Database, derived from the bioassays titled “NCI human tumor cell line growth inhibition assay” with relatively sufficient number of tested compounds (more than 16,000). Finally, filtered through 3 rules as Cheng defined [6], 37 small molecules of eligible quality were curated as the final NCI-60 dataset (in Additional file 1: Table S 1).

#### CMap dataset

In order to demonstrate the performance of the feature integration on the large-scale dataset, similarity fusion was performed on the well-known Connectivity Map dataset. [21] Justin Lamb, et al. had created the first reference collection of gene-expression profiles from cultured human cells stimulated with bioactive small molecules, together with the pattern-matching algorithm to mine these data. To date, CMap contains approximately 7,100 expression profiles representing 1,309 compounds. Some compounds with only expression profiles of HT_HG-U133A_EA Gene chips were not included in this study due to the lack of chip description information. Compared to the former NCI-60 data, the gene expression profile for a compound can also be viewed as a kind of bioactivity representation.

## Methods

### Test for NCI-60 dataset

#### Similarity matrix from two views: Bioactivity profile and molecule structure

The pairwise similarities among the 37 molecules are characterized by two similarity matrices in two views. In the view of bioactivity, similarity between two compounds is measured by the Pearson correlation coefficient of the two bioactivity profiles:

(1) $$r=\frac{n\Sigma A_{i}B_{i}-\Sigma A_{i}\Sigma B_{i}}{\sqrt{n\Sigma A_{i}^{2}-{\left(\Sigma A_{i}\right)}^{2}}\sqrt{n\Sigma B_{i}^{2}-{\left(\Sigma B_{i}\right)}^{2}}}$$

Where *n* is 37, $A_{i}$ and $B_{i}$ are the log(GI50) values in the *ith* NCI-60 cell line for the compound A and B, respectively. In the view of molecule structure, commonly-used path-based 1024-bit fingerprint of each compound is calculated via java CDK library to represent the molecular structure, and the similarity of two compounds is measured by the tanimoto-index of the two structural fingerprints:

(2) $$t=\frac{N_{\mathrm{AB}}}{N_{A}+N_{B}-N_{\mathrm{AB}}}$$

where $N_{A}\left(N_{B}\right)$ is the number of features in compound A(B), and $N_{\mathrm{AB}}$ is the number of features common to both A and B. Both of the two similarity measurements are in the interval from 0 to 1. It should be noted that correlation coefficient of bioactivity profile below 0 are assign to 0 for two reasons: (1) only very few compounds pairs have a negative correlation coefficient and the minimum is −0.2, which is not significant as an evidence of negative correlation; (2) regarding to the integration analysis of different similarity information, negative correlation brings in no better information of molecular similarity than noise. Finally, as the input for multi-view fusion [15], the two nñn similarity matrices $S=\left(S_{\mathrm{ij}}\right)$ were standardized as $S=\left(S-mean_{s}\right)/sd_{s}$ and renormalized to $P=S/\Sigma_{\mathrm{ij}}S_{\mathrm{ij}}$.

#### Fusion of similarity matrices by expectation-maximization (EM) algorithm

The fused similarity matrix is a convex combination of the L original matrices weighted by vector αwith $\Sigma_{i}a_{i}=1$ and $a_{i}\geq 0$. The advantage of this model lies that it can automatically learn the optimized proper weights for each matrix for fusion rather than arbitrary setting the values. This is achieved by an two-step alternative minimization method introduced by T. Lange and J.M. Buhmann [15]. A brief process of the two alternating steps is summarized as follow:

1. Non-negative matrix factorization:

In this study the Non-Negative Matrix Factorization (NMF) is used as one step of the minimization of cross-entropy. The target fused matrix $P\in {\left[0,1\right]}^{nñn}$ can be factorized into a product $VH^{t}$of the nñk matrices of **V** and **H**. Here the parameter k was assigned to 6 in accordance with Cheng’s number of clustering [6]. It should be noted that the selection of clustering number in a common cluster algorithm is always a non-trivial problem, however in our study, we just set the same cluster number as in the former study for an equally comparison purpose. The computational model proposed here is well extendable to tune the optimal cluster number if any pre-knowledge are unavailable. Then given the fixed weights **α**of the similarity matrices (initial **α** of (0.5, 0.5) is used and the value of **α** is updated in every iteration), we can obtain estimated and using an EM-process which minimize the cross-entropy between **P** and by updating **V** and **H** iteratively.

2. Optimizations of weights for similarity matrices by minimizing the cross-entropy:

Given the estimated factorized matrices, we minimize the cross-entropy between and $VH^{t}$

(3) $${\mathrm{Min}}_{\mathrm{\alpha VH}}\Sigma_{l}\alpha_{l}C\left(P_{l}||VH^{t}\right)$$

regarding to **α** subject to $\Sigma_{i}a_{i}=1$ and $a_{i}\geq 0$, where $C\left(P||Q\right)$ denotes the cross-entropy of **P** and **Q**, and:

(4) $$C\left(P||Q\right)=-\Sigma_{x}p\left(x\right)\log q\left(x\right)$$

Hence the second step becomes a linear program problem.

Since the solution of the linear program would tend to be too sparse that only one of the data source would be chosen to minimize the object function, which is against our intention to combine multiple data source, it is necessary to modify the object function by introducing the entropy of weight **α** so that both sparseness and informativeness could be taken into account. Since the information quantity provided by the weights vector could be measured by its entropy [15], the modified object function is:${\mathrm{Min}}_{\mathrm{\alpha VH}}\Sigma_{l}\alpha_{l}C\left(P_{l}||VH^{t}\right)-\eta H\left(\alpha \right)$ s.t. Σα=1and $\alpha_{i}\geq 0$ (5)Where $H\left(\alpha \right)$ denotes the entropy of **α**:

(5) $$H\left(\alpha \right)=-\Sigma_{i}p\left(\alpha_{i}\right)\log p\left(\alpha_{i}\right)$$

Then the second step becomes a NLP problem and can be solved with LINDO API 6.1.

#### Parameter optimization

The parameter η→∞ controls the trade-off between sparseness and informativeness. η→0 indicates that entropy will take few significance in the object function, while η→∞ indicates that information are taken as the most important factor in the object function, and the weights of different source are evenly distributed. Tuning η is a non-trivial work. In the previous work the sampling-based assessment of η is not suitable for clustering of small size objects (like the NCI-60 dataset in this study). In our study a leave-one-out stability assessment was used to assign a proper value of η. The ideal η is expected to render better stability when a clustering is performed. For NCI-60 dataset, we performed 37 times leave-one-out sampling for clustering of the whole data, and each time 36 compounds were selected. A series of η ranging from 0.001 to 1000 were used in the fusion model. Two parameters were used as the evaluation of the performance regarding to different η value, as listed in the following. It should be noted that other measurements can also be adopted to tune the η value in clustering, which will be generally consistent to these two measurements and will not be discussed here:

1. Average mean disagreement (AMD)

AMD is defined as the average value of the mean disagreement among the 37 subgroups. Given the clustering results $Y\in {\left\{1,2,...,k\right\}}^{n}$ by cutting the clustering tree into k class with by cutting the clustering tree into k class with $k\in \left[2,15\right]$, the AMD is defined as:

(6) $$\mathrm{AMD}=\frac{1}{s}\Sigma_{1}^{s}mean_{Y,Y'\in Y}\Sigma_{i=1}^{n}I\left\{y_{i}\neq y{'}_{i}\right\}$$

Where *S* is the number of subgroups, Y,Y' are two clustering result with equal k value, $y_{i}$ and $y{'}_{i}$ are class labels of element i in two results respectively, $I_{\left\{A\right\}}$ is the indicator function of expression A. The η value with a lower AMD in the clustering result is considered as a good parameter.

2. Average Dunn’s Index (ADI)

ADI is used to describe the partition quality in a clustering result. Dunn’s index is defined as the ratio of the minimal interclass distance and maximal intra-class distance. Higher Dunn’s index indicates better validity of partition. For NCI-60 dataset we calculate the average Dunn’s index regarding to a range of class number $k\in \left[2,15\right]$ as defined in AMD. The η that obtains a high Dunn’s index with low variance could be considered as a proper estimation.

#### Fused similarity matrix

After estimating the sparseness controlling parameter, the alternative minimization steps were repeated on the whole dataset. Final weights vector α was calculated and the fused similarity matrix can be obtained as the convex combination of the two original similarity matrices with the weights **α**.

#### Compound-target interaction analysis

A compound-target interaction network can be constructed via target annotation in the PubChem BioAssay database. In general, one compound (identified by CID) was linked to a target protein (identified by NCBI protein ID, or GI) if this compound was tested active in the bioassay which was specified with the protein target. All the target annotation and activity information was retrieved from PubChem BioAssay database via E-Utilities tool. The interaction network was constructed and visualized by using the Cytoscape (version 2.7.0) [22], containing 37 compound nodes and 138 target nodes (in Additional file 1: Table S 2).

We proposed a quantitative method to analysis the relationship between compound similarity and their protein targets. This method is based on the concerning that compounds which have similar features, either structural or biological, tend to share common protein target. Based on such assumption, it is nature to build a connection between the quantitative similarity between compounds and the common target number within a group of compounds. And it is obvious to conclude that the common target number in a cluster derived by a clustering algorithm is an efficient measurement to measure the quality of the similarity adopted for this clustering. The larger common target number obtained in a cluster generally reveals a more reasonable similarity adopted. Followed by this strategy, in our study the compound-target interaction network was modified by taking out all the target nodes by linking two compound nodes together if they have a common protein target. The modifying process was carried out using Pajek [23]. And an average degree within a cluster was presented, which is calculated as an efficient measurement of the common target number in this cluster:

(7) $$D=\frac{1}{n}\Sigma_{j=1}^{n}D_{j}$$

Where $D_{j}$ is the degree of node j in the graph and *n* is the number of nodes. The degree analysis was accomplished by using igraph package (version 0.5.1) [24] in R (version 2.12.0) [25].

### Similarity fusion on large-scale CMAP dataset

In order to demonstrate the performance of the feature integration on the large-scale dataset, similarity fusion was performed on the CMap dataset. In this study, in order to generate pair-wise relationship among all the compounds, Gene Ontology (GO) fingerprint [26], which is presented in our previous study as a well-defined bioactivity representation, was adopted to combine all the expression profiles of one compound and reduce the high dimensions and noises in the microarray data. This descriptor was used to describe drug in a biological activity view. Similarly, the same structural fingerprint as used for NCI-60 data was used here to describe drug in a compound structure view.

The fusion of structural fingerprint and GO-fingerprint similarity matrices was performed following the same workflow aforementioned for NCI-60 data. And the detailed parameter optimization will not be discussed here. Considering that clustering result of large scale dataset cannot be analysed straightforwardly as the former 37-compound dataset, two typical HDAC and HSP90 inhibitors, which was used as the examples in Lamb’s work, were chosen as the queries to validate our fusion method from the perspective of virtual drug screen. For each query, the ranks of similarity searching derived by the fused similarity were compared to that with only single view, and the targets of top-ranked compounds with similarity above 0.5 to queries were also analysed for further discussion.

## Results and discussions

### Test results for NCI-60 dataset

#### Assessment of the sparseness-controlling parameter for NCI-60 data

**Figure 2:**
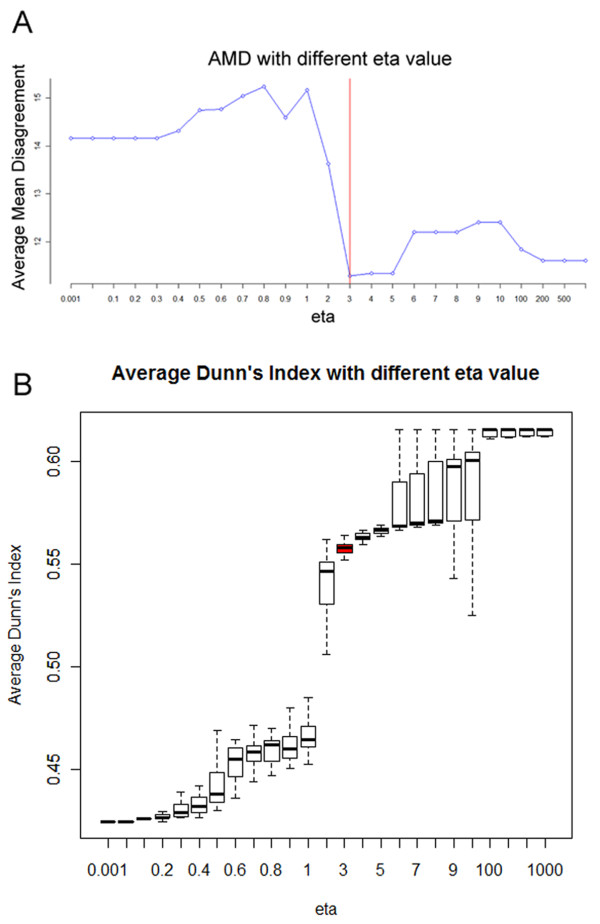
**Parameter optimization.** Average Mean Disagreement(AMD) and Average Dunn’s Index(ADI) with different η value. When η = 3, AMD and ADI was marked with red color.

For the stability assessment, η was chosen in a range as (0.001, 0.01, 0.1, 0.2, 0.3, 0.4, 0.5, 0.6, 0.7, 0.8, 0.9, 1, 2, 3, 4, 5, 6, 7, 8, 9, 10, 100, 200, 500, 1000). It should be noted that if η is smaller than 0.5, extreme large weight would be added on one of the two original similarity matrices (larger than 0.99), while η larger than 10 will generally separate the weight evenly between the two matrices, i.e. 0.5 for each. After the 37 times leave-one-out subgroup clustering, two parameters, AMD and ADI were calculated as the evaluations of the clustering quality (Figure 2).

As shown in Figure 2A, the Average Mean Disagreement reached the lowest value when η=3. Furthermore, the Average Dunn’s Index indicated the validity of the clustering. As shown in Figure 2B, the ADI grew gradually when η increased below 3. The decreasing variance suggested an accretive clustering quality. It should be noted that when η=3 ADI has a sharp rise, while after that the trend of growing has become attenuated. Later calculation of weights **α** reveals that η lower than 3 or greater than 100 will tend to give biased weights to the two matrix, i.e. either $\alpha =\left[0,1\right]$ or $\alpha =\left[0.5,0.5\right]$. In summary, given the best value of η in AMD, and a relative high value in ADI, it is reasonable to choose η=3 as a proper estimation to control the sparseness.

#### Clustering result

**Figure 3:**
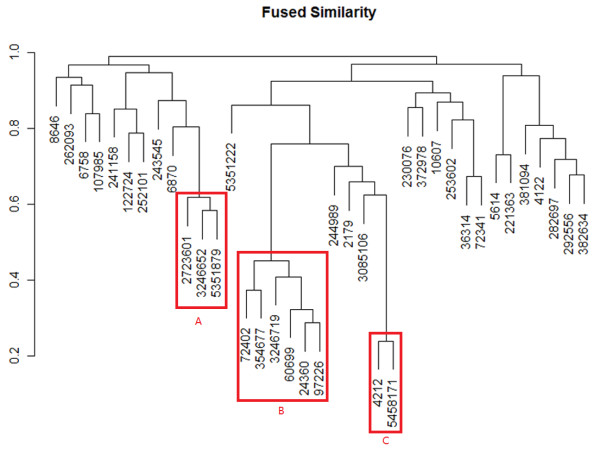
**Clustering result using fused similarity.** Numbers represent Pubchem Compound ID (CID). The value of distance d transformed from similarity $\text{s:d}=\sqrt{1-s}$ is shown in the left. Three distinct classes was marked with a red box and named as: **A** [CID: 2723601, 3246652, 6351879]; **B **[CID: 24360, 60699, 72402, 354677, 5351879]; **C** [CID: 4212, 5458171].

A hierarchy clustering result for the 37 compounds based on fused similarity is shown in Figure 3. It should be noted that there exist several differences on the structure of the hierarchy clustering tree compared to single-view similarity clustering, as shown in Cheng’s work (The previous clustering results of single-view similarity are shown in Additional file 1: Figure S 1). Generally speaking, using correlation of bioactivity profile instead of Euclidean distance helps to find a new member in one cluster (referred to as cluster B in Cheng’s work [6]), and our fused similarity clustering result combines distinct clusters that exist in different single-view clustering separately. It should be noted that clusters with low fused similarity (with a distance above 0.7 in this study) have neither significant structural nor biological resemblance, hence no detailed analysis was presented. In the following part 3 interesting findings for NCI-60 data from the multi-view clustering were discussed compared to the clustering with only bioactivity profiles or structural fingerprints respectively.

##### Overall average common target number

**Figure 4:**
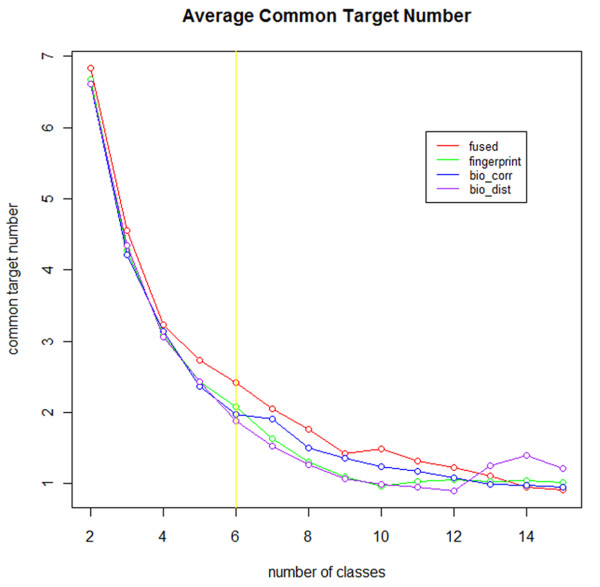
**Average common target number of the clustering result.** The hierarchy clustering tree was cut into a range of classes. The common target obtained by fused similarity, structural similarity, bioactivity profile similarity and bioactivity profile Euclidean distance were represented by red, green, blue and purple lines respectively. The value of the default class number 6 was marked with a yellow line.

As shown in Figure 4, the *x* axis is the number of classes the hierarchy tree was cut into; the y axis was the average common compound target number within one class. It is apparent that the common target number would decrease as the member within each class drops when class number increases, since the number of objects in each class decreased. The common target obtained by fused similarity, structural similarity, bioactivity profile similarity and bioactivity profile Euclidean distance were represented by red, green, blue and purple lines respectively. It is interesting to find that in general the common target number obtained by fused similarity is larger than those obtained by the other two single-view similarities, which indicates that the multi-view data representation provides a better similarity measurement and clustering validity in target-specific compound analysis compared to single-view clustering.

**Figure 5:**
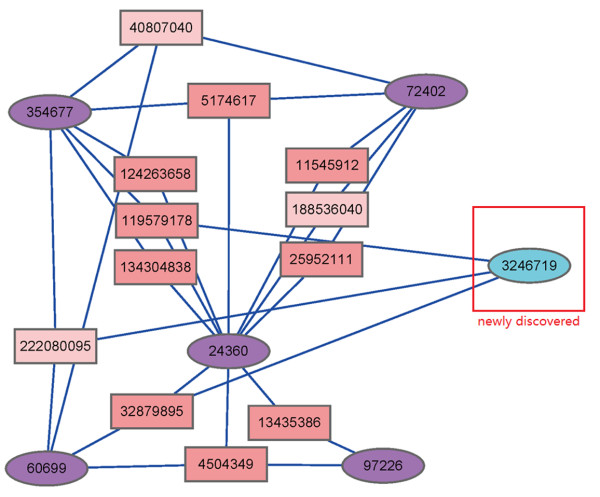
**Compound-targets network of clustering.** Protein Targets are represented in rectangle shape, and the corresponding compounds are represented in eclipse shape in cluster B. Compounds in previous cluster B were marked in purple. The newly discovered class member using fused similarity was marked in blue.

##### Highly similar structure as complement of bioactivity profiles

**Figure 6:**
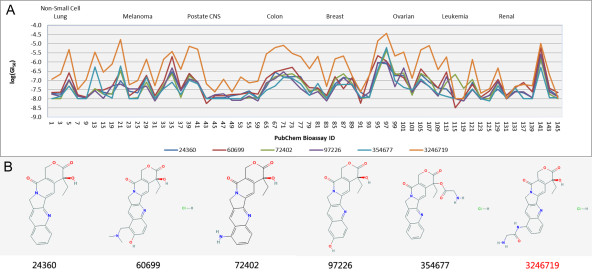
**Bioactivity profile(A) and compound structure (B) of the 6 compounds in cluster B.** Bioactivity profile and compound structure of the 6 compounds in cluster B were presented in Figure 6A and 6B respectively.

In the hierarchical tree a cluster (Cluster B in Figure 3) with 6 compounds [CID: 72402, 354677, 3246719, 60699, 24360 and 97226] is distinctive in the final clustering result. Among the 6 compounds, 5 of them correspond to the Cluster B in the previous clustering achieved only with bioactivity profiles [6]. It is quite interesting that the one excluded in the single-view clustering was finally introduced into this cluster when the structural information and bioactivity profile information were considered in an integrated way. An insight into the bioactivity profiles reveals that compound [CID: 3246719] was excluded in the former study for a probable reason that its bioactivity profile shifts above the other 5 profiles (Figure 6A), but keeps the similar shape of the profile curve. By further comparing their structures, it is clearly to observe the high similarity among the 6 compounds (Figure 6B). It is possible to reason that the intrinsic similar structures of the 6 compounds results in the similar pattern of bioactivity profiles, i.e. similar chance to function in the compound-target network, and the up-shift dosage of the outlier compound above other bioactivity profiles will influence little on its functions related to specific target. However, with only bioactivity profile distance measurement, such information may be lost by ignoring structural resemblance and corresponding bioactivity correlation. It can be seen that there exist an approximately 1 order of magnitude difference between the GI50 of compound [CID:3246719] and the other 5 compounds. Therefore their bioactivity profiles will varied significantly while the correlation remains relative large.

Further compound-target interaction analysis on the protein targets within this cluster shows that the compound CID: 3246719 shares common protein target with other members in the group, while this compound was missing in the previous compound-target network [6] as shown in Additional file 1: Figure S 1A. It can be seen that there’re three protein target linked to compound CID: 3246719 [GI: 119579178, 222080095 and 3287985] (Figure 5). All the three protein targets are shared in the former single-view clustering. It is indicated that by using multi-view similarity analysis a missing group member was discovered by introducing extra structural information. It is evident that compounds sharing similar structural features and bioactivity profiles simultaneously will give bonus to the performance in a similarity-based search. In addition, other two compounds (Cluster C, [CID: 4212 and 5458171]) can be another good examples. These two compounds are significantly similar both in structure and bioactivity profiles (Additional file 1: Figure S 3); hence they get a notably high similarity in the hierarchy tree (Figure 1).

##### Highly similar bioactivity profiles as complement of moderately similar structure

Another cluster composed of three compounds [CID: 2723601, 3246652 and 5351879] are noteworthy to explain in the fused hierarchy clustering tree. If we only measure the compound similarity with structural information, we can see that there are relatively less similar. However, when combined with the bioactivity information, these three compounds successfully merged into the first cluster during hierarchical clustering [6]. (Additional file 1: Figure S 2). Target interacting analysis reveals that these three compounds share a common target [GI: 4504349], indicating a potential common function in biological process. It is notable that certain fragment of the compounds, thioguanine in this example, instead of the complete structure, is essential in a binding event. Therefore when compounds that bind to a common target exhibit only relatively low overall structural similarity, it could be a good complementary to introduce the bioactivity profiles to suggest a more strong correlation with target binding potent. Such advantage of multi-view similarity assessment could be remarkable when no prior knowledge about either specific functional fragment or target is available.

### Drug virtual screen based on fused similarity of CMap dataset

Firstly, trichostatin A (TSA), a typical Histone deacetylases (HDAC) inhibitor [27], was used as the query of similarity searching based on fused similarity, GO fingerprint and structural fingerprint respectively. The top 10 ranking results were listed in Table 1. It is very interesting that among all the ranking compounds, vorinostat and scriptaid, two strong HDAC inhibitors [28,29] were successfully retrieved in the top 2 (1^st^ and 2^nd^ of the 1267 compounds) using fused similarity. As shown in Table 1, vorinostat and scriptaid ranks as the top two candidates in the view of GO fingerprint, which indicates their similar expression profiles. However in the view of structural fingerprint, vorinostat and scriptaid were ranked 83^rd^ and 295^th^ respectively. Further analysis on the weighing scheme reveals that this is reasonable since the optimized fusion method successfully give a larger weight on the information-rich GO fingerprint.

**Table 1:** Similarity search based on fused similarity: HDAC inhibitors

	**Fused similarity**		**GO fingerprint**		**Structural fingerprint**	
**Rank**	**Compound**	**Similarity**	**Compound**	**Similarity**	**Compound**	**Similarity**
**1**	**vorinostat**	**0.5831**	**vorinostat**	0.6842	chlorambucil	0.3768
**2**	**scriptaid**	**0.5308**	**scriptaid**	0.6293	mifepristone	0.3512
3	mycophenolic acid	0.3849	thapsigargin	0.4567	IC-86621	0.3289
4	thapsigargin	0.3825	mycophenolic acid	0.4444	menadione	0.3037
5	rifabutin	0.3719	rifabutin	0.4372	ciclopirox	0.2941
6	penbutolol	0.3630	ouabain	0.4245	3-hydroxy-DL-kynurenine	0.2903
7	benzethonium chloride	0.3575	cephaeline	0.4196	crotamiton	0.2857
8	cephaeline	0.3566	penbutolol	0.4162	fenbufen	0.2766
9	GW-8510	0.3562	GW-8510	0.4144	N-phenylanthranilic acid	0.2761
10	flunixin	0.3530	benzethonium chloride	0.4081	bupropion	0.2701

Trichostatin A (TSA) was used as the query compound.

Top-ranked HDAC inhibitors were marked in bold.

More interesting results were discovered on the similarity searching of geldanamycin, an HSP90 inhibitor [30]. (Table 2) It can be seen that tanespimycin and alvespimycin, both the derivants of geldanamycin, which are also typical HSP90 inhibitors [31,32], were ranked at 1st and 2 rd place using fused similarity. In addition, monorden, which is another common HSP90 inhibitor [33] was ranked in top 10 (rank 8^th^). However, when using only GO fingerprint and Structure fingerprint, monorden was ranked 10^th^ and 123^rd^ respectively. More interestingly, 15-delta prostaglandin J2 (15d-PGJ2), suggested to exert anti-inflammatory effects *in vivo*[34], ranked 3^rd^ in the fusion similarity searching. Further literature research on its target indicates that HSP90 is a target for modification by 15d-PGJ2 in renal mesangial cells. [35] This result shows that by assigning balanced weights to the two views, our fusion method successfully picked out this newly discovered HSP90 inhibitor, thus demonstrated the fused similarity provides an effective quantitative assessment of drug-target relationship.

**Table 2:** Similarity search based on fused similarity: HSP90 inhibitors

	**Fused similarity**		**GO fingerprint**		**Structural fingerprint**	
**Rank**	**Compound**	**Similarity**	**Compound**	**Similarity**	**Compound**	**Similarity**
1	**tanespimycin**	**0.7481**	**tanespimycin**	0.7262	**tanespimycin**	0.8253
2	**alvespimycin**	**0.6155**	**15-delta prostaglandin J2**	0.5914	**alvespimycin**	0.7880
3	**15-delta prostaglandin J2**	**0.5111**	**alvespimycin**	0.5667	securinine	0.4055
4	thiostrepton	0.4894	sodium phenylbutyrate	0.5579	sirolimus	0.3826
5	scopolamine N-oxide	0.4785	scopolamine N-oxide	0.5568	tacrolimus	0.3723
6	monorden	0.4737	thiostrepton	0.5455	meclocycline	0.3524
7	cefsulodin	0.4693	nordihydroguaiaretic acid	0.5375	rifabutin	0.3516
8	tetracycline	0.4603	monorden	0.5366	chlortetracycline	0.3508
9	sodium phenylbutyrate	0.4550	prochlorperazine	0.5326	demeclocycline	0.3508
10	LY-294002	0.4544	cefsulodin	0.5294	5707885	0.3471

Geldanamycin was used as the query compound.

Top-ranked HSP90 inhibitors were marked in bold.

## Conclusions

A multi-view clustering method was introduced to discover a more robust correlation between fused multi-view similarity and compound-target interacting pattern. By using a similarity-based optimization and fusion model, a hierarchy clustering integrated with both structural and bioactivity profile information was presented on the NCI-60 dataset. It is interesting that comparing to single view analysis, the overall common target number within fused classes has been promoted by integrating information from two views, which indicated a more robust and efficient representation of compound related to specific target. Analysis of compound-target interaction network shows that fusion of data source from different views enhances similar compound discovery, leading to a more comprehensible assessment of target-binding potent. Further analysis in certain classes with high fused similarity shows that the mutual complement of the two views can lead to the discovery of missing similar compound with only one view. A further large-scale similarity searching on the CMap data based on the fused similarity also obtained a better ranking results compared to that of single-view for two inhibitors as queries, thus indicate the potential use of our quantitative similarity fusion in virtual drug screen. In summary, our findings are interesting for the following reasons: Firstly, both the bioactivity profiles and structural fingerprint lack to be a completely direct indicator of interaction, i.e. only partial features instead of overall characterization from either view are essential in a binding event. Hence by integrating potentially correlating features from both views to maximize the utility of available data source, a robust similarity assessment could be achieved without prior knowledge about the detail relationship between target-binding rules and compound features. Secondly, the fusion method in this study provides an extendable framework of integrating multi-view data. Fusion process is applicable to various situations when more than two data sources are available. A comprehensive assessment of the similarity can be achieved in virtual drug screening when various potential pharmacological properties of compounds are integrated.

## Competing interests

The authors declare that they have no competing interests.

## Authors' contributions

ZC and QL conceived and participated in the design of the study. TX and RZ performed the data collection and processing. TX and QL performed the whole experiments and drafted the manuscript. All authors read and approved the final manuscript.

## Supplementary Material

Additional file 1 **Additional tables and figures were saved in the word file entitled “supplementary.doc”, containing the 37 compounds CID list, target information and other clustering results for the NCI-60 dataset, etc.Additional tables and figures were saved in the word file entitled “supplementary.doc”, containing the 37 compounds CID list, target information and other clustering results for the NCI-60 dataset, etc.** Click here for file
