# Supplementary material for: Quantitatively integrating molecular structure and bioactivity profile evidence into drug-target relationship analysis
Source: BMC Bioinformatics. 2012 May 4;13:75. doi: 10.1186/1471-2105-13-75 (PMC3528629; doi:10.1186/1471-2105-13-75)
Supplement: Additional file 1 — Additional tables and figures were saved in the word file entitled “supplementary.doc”, containing the 37 compounds CID list, target information and other clustering results for the NCI-60 dataset, etc.Additional tables and figures were saved in the word file entitled “supplementary.doc”, containing the 37 compounds CID list, target information and other clustering results for the NCI-60 dataset, etc. [file 1471-2105-13-75-S1.doc]

Quantitatively Integrating Molecular Structure and Bioactivity Profile Evidence into Drug-Target Relationship Analysis

Tianlei Xu^1^, Ruixin Zhu^1^ Qi Liu^1,*^and Zhiwei Cao^1,*^

^1^Department of Bioinformatics, Tongji University, 200092, Shanghai, China.

**Table S1.**PubChem ID of the 37 Compound used in this study

| No. | CID |
| --- | --- |
| 1 | 2179 |
| 2 | 4122 |
| 3 | 4212 |
| 4 | 5614 |
| 5 | 6758 |
| 6 | 6870 |
| 7 | 8646 |
| 8 | 10607 |
| 9 | 24360 |
| 10 | 36314 |
| 11 | 60699 |
| 12 | 72341 |
| 13 | 72402 |
| 14 | 97226 |
| 15 | 107985 |
| 16 | 122724 |
| 17 | 221363 |
| 18 | 230076 |
| 19 | 241158 |
| 20 | 243545 |
| 21 | 244989 |
| 22 | 252101 |
| 23 | 253602 |
| 24 | 262093 |
| 25 | 282697 |
| 26 | 292556 |
| 27 | 354677 |
| 28 | 372978 |
| 29 | 381094 |
| 30 | 382634 |
| 31 | 2723601 |
| 32 | 3085106 |
| 33 | 3246652 |
| 34 | 3246719 |
| 35 | 5351222 |
| 36 | 5351879 |
| 37 | 5458171 |

**Table S2.**Compound (CID) and target (GI)

| **CID** | **GI** |
| --- | --- |
| 2179 | 4826730 |
| 2179 | 13959709 |
| 2179 | 18249941 |
| 2179 | 73915100 |
| 2179 | 187960037 |
| 4122 | 7705682 |
| 4122 | 42741659 |
| 4122 | 73915100 |
| 4122 | 116241283 |
| 4212 | 4557365 |
| 4212 | 4758356 |
| 4212 | 6980812 |
| 4212 | 13435386 |
| 4212 | 18249941 |
| 4212 | 21620132 |
| 4212 | 30027657 |
| 4212 | 38156699 |
| 4212 | 40807040 |
| 4212 | 41055989 |
| 4212 | 66932916 |
| 4212 | 73915100 |
| 4212 | 84028058 |
| 4212 | 90903231 |
| 4212 | 92096784 |
| 4212 | 120407068 |
| 4212 | 120660324 |
| 4212 | 187960037 |
| 4212 | 282403581 |
| 5614 | 4826730 |
| 5614 | 7705682 |
| 5614 | 8659577 |
| 5614 | 10954339 |
| 5614 | 13435386 |
| 5614 | 13699818 |
| 5614 | 18249941 |
| 5614 | 30582681 |
| 5614 | 32879895 |
| 5614 | 38016895 |
| 5614 | 38156699 |
| 5614 | 40807040 |
| 5614 | 41055989 |
| 5614 | 68476498 |
| 5614 | 73915100 |
| 5614 | 83774548 |
| 5614 | 92096784 |
| 5614 | 120407068 |
| 5614 | 120660324 |
| 5614 | 187960037 |
| 5614 | 219518789 |
| 5614 | 222080095 |
| 5614 | 285809906 |
| 5614 | 285814664 |
| 6758 | 4503219 |
| 6758 | 4826730 |
| 6758 | 7705682 |
| 6758 | 13435386 |
| 6758 | 42741659 |
| 6758 | 73915100 |
| 6758 | 187960037 |
| 6870 | 92096784 |
| 6870 | 188536040 |
| 8646 | 4504349 |
| 8646 | 6323930 |
| 8646 | 32879895 |
| 8646 | 40807040 |
| 10607 | 112822 |
| 10607 | 257380 |
| 10607 | 548814 |
| 10607 | 13272532 |
| 10607 | 13435386 |
| 10607 | 20070193 |
| 10607 | 21392848 |
| 10607 | 34577122 |
| 10607 | 40807040 |
| 10607 | 62740231 |
| 10607 | 68299797 |
| 10607 | 119579178 |
| 10607 | 124263658 |
| 10607 | 223468676 |
| 24360 | 486173 |
| 24360 | 4503219 |
| 24360 | 4504349 |
| 24360 | 4826730 |
| 24360 | 5174617 |
| 24360 | 11545912 |
| 24360 | 12644118 |
| 24360 | 13435386 |
| 24360 | 25952111 |
| 24360 | 32879895 |
| 24360 | 38788193 |
| 24360 | 73915100 |
| 24360 | 119579178 |
| 24360 | 120660324 |
| 24360 | 124263658 |
| 24360 | 134304838 |
| 24360 | 188536040 |
| 36314 | 10954339 |
| 36314 | 55958172 |
| 60699 | 510901 |
| 60699 | 4504349 |
| 60699 | 17507875 |
| 60699 | 20336335 |
| 60699 | 31881630 |
| 60699 | 32879895 |
| 60699 | 40807040 |
| 60699 | 56550039 |
| 60699 | 71274194 |
| 60699 | 78070770 |
| 60699 | 139472804 |
| 60699 | 222080095 |
| 72341 | 31881630 |
| 72402 | 4505931 |
| 72402 | 5174617 |
| 72402 | 11545912 |
| 72402 | 25952111 |
| 72402 | 32307152 |
| 72402 | 40807040 |
| 72402 | 45219878 |
| 72402 | 46367787 |
| 72402 | 188536040 |
| 97226 | 4504349 |
| 97226 | 13435386 |
| 97226 | 44888968 |
| 107985 | 5174617 |
| 107985 | 15929025 |
| 107985 | 38788193 |
| 107985 | 62740231 |
| 107985 | 119579178 |
| 107985 | 124263658 |
| 107985 | 160707929 |
| 107985 | 167013344 |
| 107985 | 188536040 |
| 122724 | 31881630 |
| 221363 | 4503219 |
| 221363 | 31881630 |
| 221363 | 32879895 |
| 230076 | 90903231 |
| 230076 | 188536040 |
| 241158 | 4325211 |
| 241158 | 5174513 |
| 241158 | 30582681 |
| 241158 | 31542939 |
| 241158 | 31881630 |
| 241158 | 158515318 |
| 243545 | 4503219 |
| 243545 | 73915100 |
| 244989 | 15149312 |
| 244989 | 15675770 |
| 244989 | 90111653 |
| 244989 | 119579178 |
| 244989 | 222080095 |
| 252101 | 21359816 |
| 252101 | 24987805 |
| 252101 | 31881630 |
| 252101 | 32879895 |
| 252101 | 73915100 |
| 252101 | 158515318 |
| 253602 | 994798 |
| 253602 | 38156699 |
| 253602 | 40807040 |
| 253602 | 62740231 |
| 253602 | 111034851 |
| 253602 | 124513266 |
| 253602 | 167013344 |
| 253602 | 219518789 |
| 253602 | 285809906 |
| 262093 | 4502003 |
| 262093 | 4503219 |
| 262093 | 4503895 |
| 262093 | 4557365 |
| 262093 | 4581413 |
| 262093 | 4758484 |
| 262093 | 4826730 |
| 262093 | 6166485 |
| 262093 | 7669492 |
| 262093 | 7705682 |
| 262093 | 7706645 |
| 262093 | 8659577 |
| 262093 | 9628427 |
| 262093 | 10954339 |
| 262093 | 13435386 |
| 262093 | 13699818 |
| 262093 | 15149312 |
| 262093 | 15431328 |
| 262093 | 15724400 |
| 262093 | 18249941 |
| 262093 | 21392848 |
| 262093 | 21595511 |
| 262093 | 26667227 |
| 262093 | 30582681 |
| 262093 | 31542939 |
| 262093 | 31881630 |
| 262093 | 38016895 |
| 262093 | 38156699 |
| 262093 | 40805836 |
| 262093 | 40807040 |
| 262093 | 46395496 |
| 262093 | 55958172 |
| 262093 | 66932916 |
| 262093 | 72386991 |
| 262093 | 73915100 |
| 262093 | 92096784 |
| 262093 | 120407068 |
| 262093 | 120538355 |
| 262093 | 120660324 |
| 262093 | 122920737 |
| 262093 | 155969707 |
| 262093 | 187960037 |
| 262093 | 188536040 |
| 262093 | 189491771 |
| 262093 | 216548487 |
| 262093 | 222080095 |
| 262093 | 262118306 |
| 262093 | 281307097 |
| 262093 | 285809906 |
| 262093 | 285814664 |
| 282697 | 119579178 |
| 282697 | 124263658 |
| 292556 | 4503131 |
| 292556 | 18959272 |
| 292556 | 119579178 |
| 354677 | 5174617 |
| 354677 | 15929025 |
| 354677 | 40807040 |
| 354677 | 119579178 |
| 354677 | 124263658 |
| 354677 | 134304838 |
| 354677 | 222080095 |
| 372978 | 4557365 |
| 372978 | 90903231 |
| 372978 | 188536040 |
| 381094 | 4759012 |
| 381094 | 6166485 |
| 381094 | 18959272 |
| 381094 | 47123300 |
| 381094 | 54112432 |
| 381094 | 119579178 |
| 381094 | 262118306 |
| 381094 | 270133071 |
| 382634 | 4503131 |
| 2723601 | 4504349 |
| 2723601 | 31542939 |
| 2723601 | 40807040 |
| 2723601 | 67191027 |
| 2723601 | 122920737 |
| 3085106 | 42741659 |
| 3246652 | 4503219 |
| 3246652 | 4504349 |
| 3246652 | 4557729 |
| 3246652 | 10092619 |
| 3246652 | 13699818 |
| 3246652 | 30582681 |
| 3246652 | 31542939 |
| 3246652 | 32879895 |
| 3246652 | 40807040 |
| 3246719 | 10954339 |
| 3246719 | 32879895 |
| 3246719 | 85986601 |
| 3246719 | 119579178 |
| 3246719 | 222080095 |
| 5351222 | 510901 |
| 5351222 | 4557365 |
| 5351222 | 4758208 |
| 5351222 | 4758484 |
| 5351222 | 6980812 |
| 5351222 | 7582271 |
| 5351222 | 15149312 |
| 5351222 | 16758752 |
| 5351222 | 21595511 |
| 5351222 | 23893623 |
| 5351222 | 27807367 |
| 5351222 | 45219878 |
| 5351222 | 45357394 |
| 5351222 | 46367787 |
| 5351222 | 49168602 |
| 5351222 | 56790945 |
| 5351222 | 78070770 |
| 5351222 | 114881106 |
| 5351222 | 139424501 |
| 5351222 | 148539876 |
| 5351222 | 189491771 |
| 5351222 | 221046486 |
| 5351222 | 270133071 |
| 5351879 | 4504349 |
| 5351879 | 73915100 |
| 5458171 | 23893623 |
| 5458171 | 23943882 |
| 5458171 | 40807040 |
| 5458171 | 49574532 |
| 5458171 | 54112432 |
| 5458171 | 56790945 |
| 5458171 | 82503229 |
| 5458171 | 117938328 |
| 5458171 | 139472804 |
| 5458171 | 148539876 |
| 5458171 | 153217451 |
| 5458171 | 216409728 |
| 5458171 | 222080095 |

**Fig. S1.** Hierarchy Clustering Tree Structure Comparison


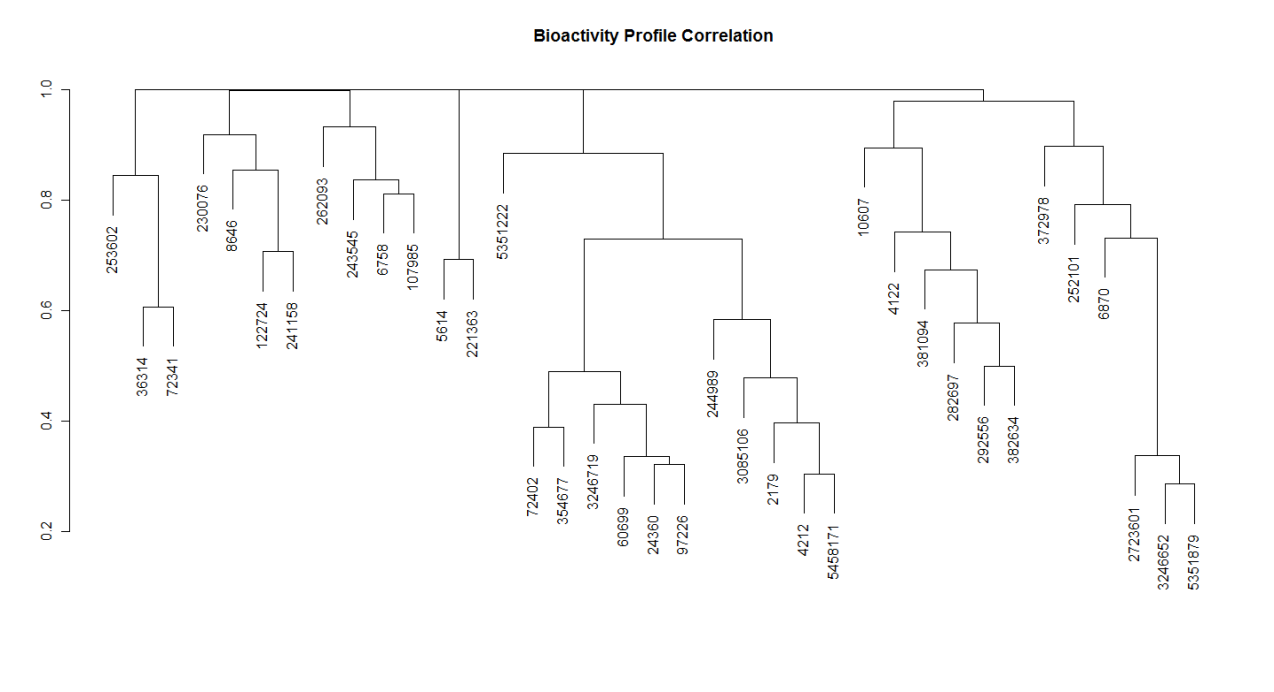


**A** Clustering result using Correlation of Bioactivity Profile


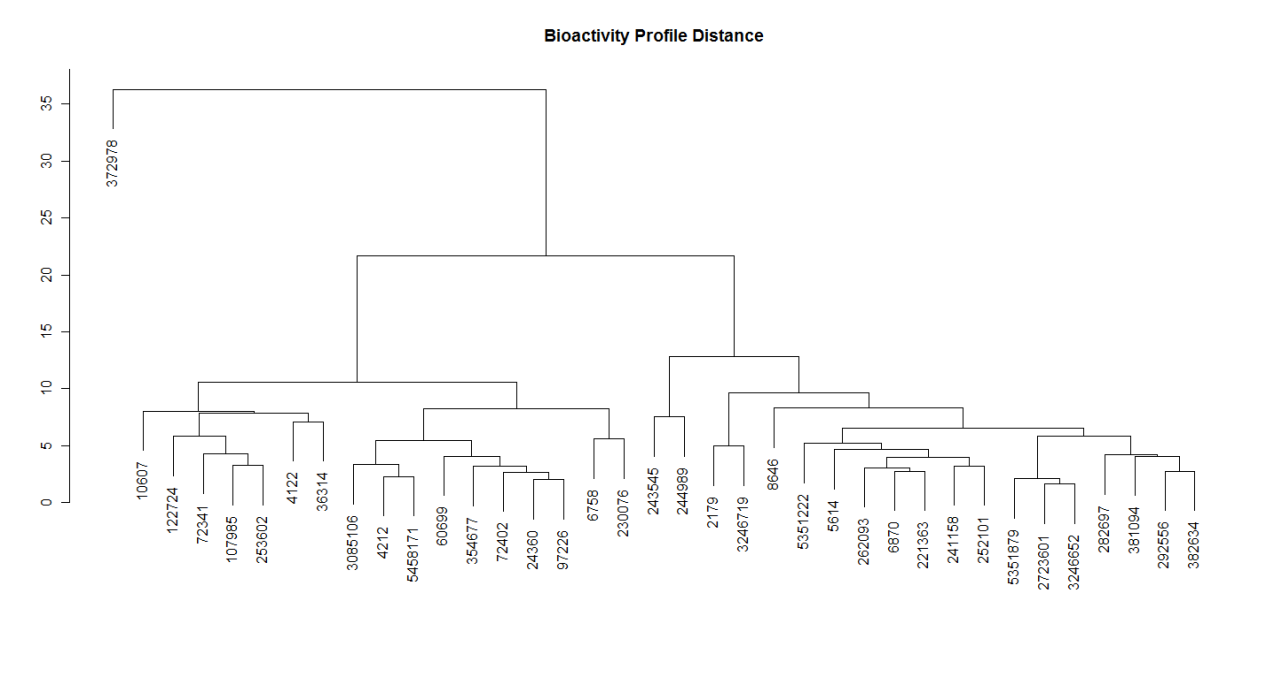


**B Clustering result using Distance of Bioactivity Profile**


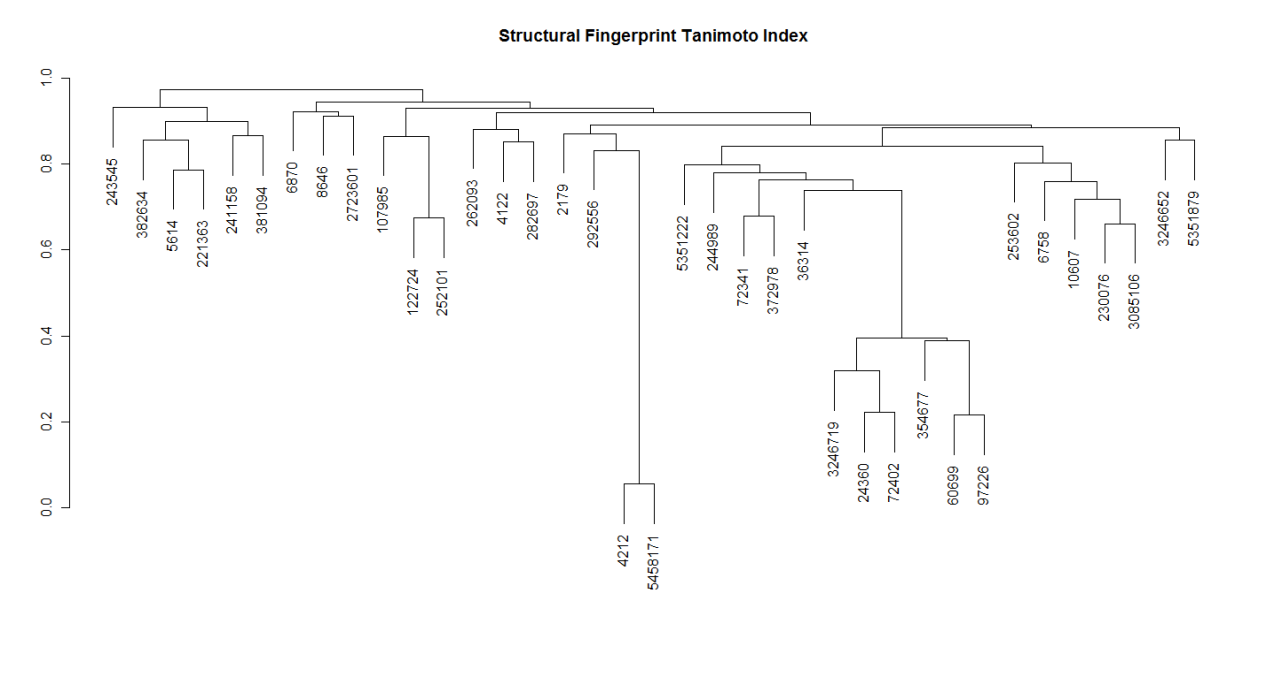


**C** Clustering result using Tanimoto Index of Structural Fingerprint


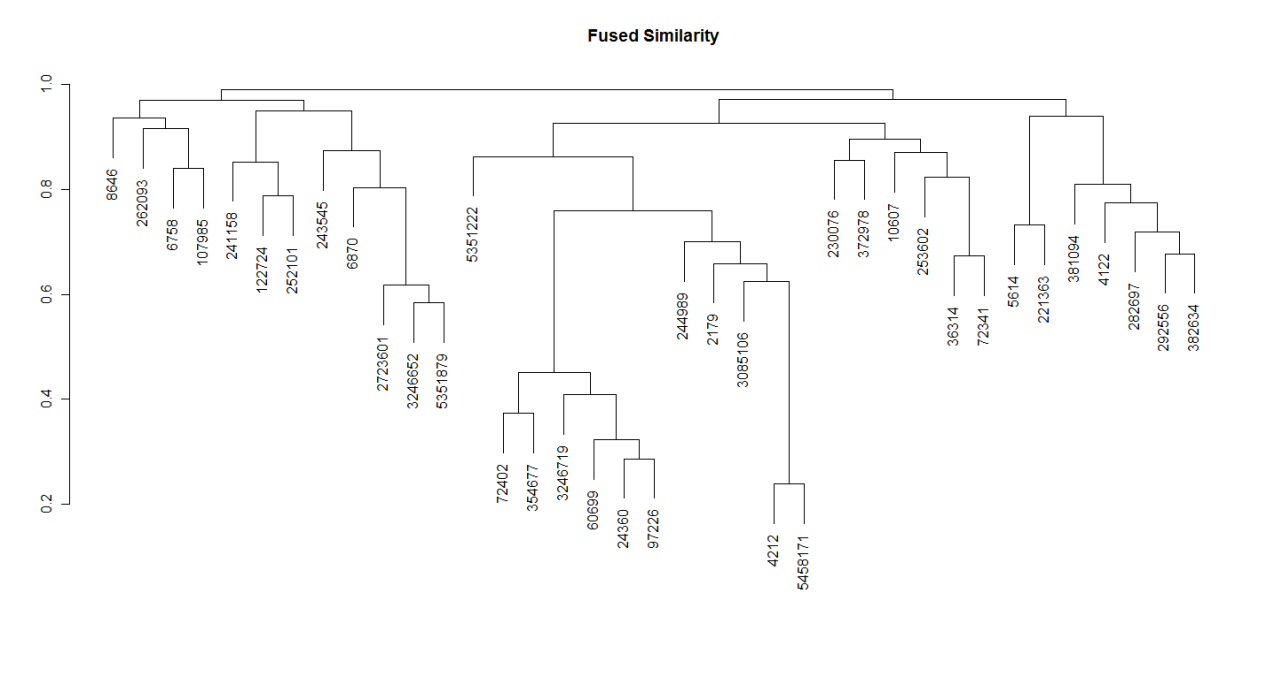


**D** Clustering result using Fused Similarity.

Using correlation of bioactivity profile instead of Euclidean distance helps to find a new member in one cluster (referred to as cluster B in Cheng’s work ), and the fused similarity clustering result combines distinct clusters that exist in different single-view clustering results separately.

**Fig. S2.** Bioactivity profile, molecular structure and target information of cluster A


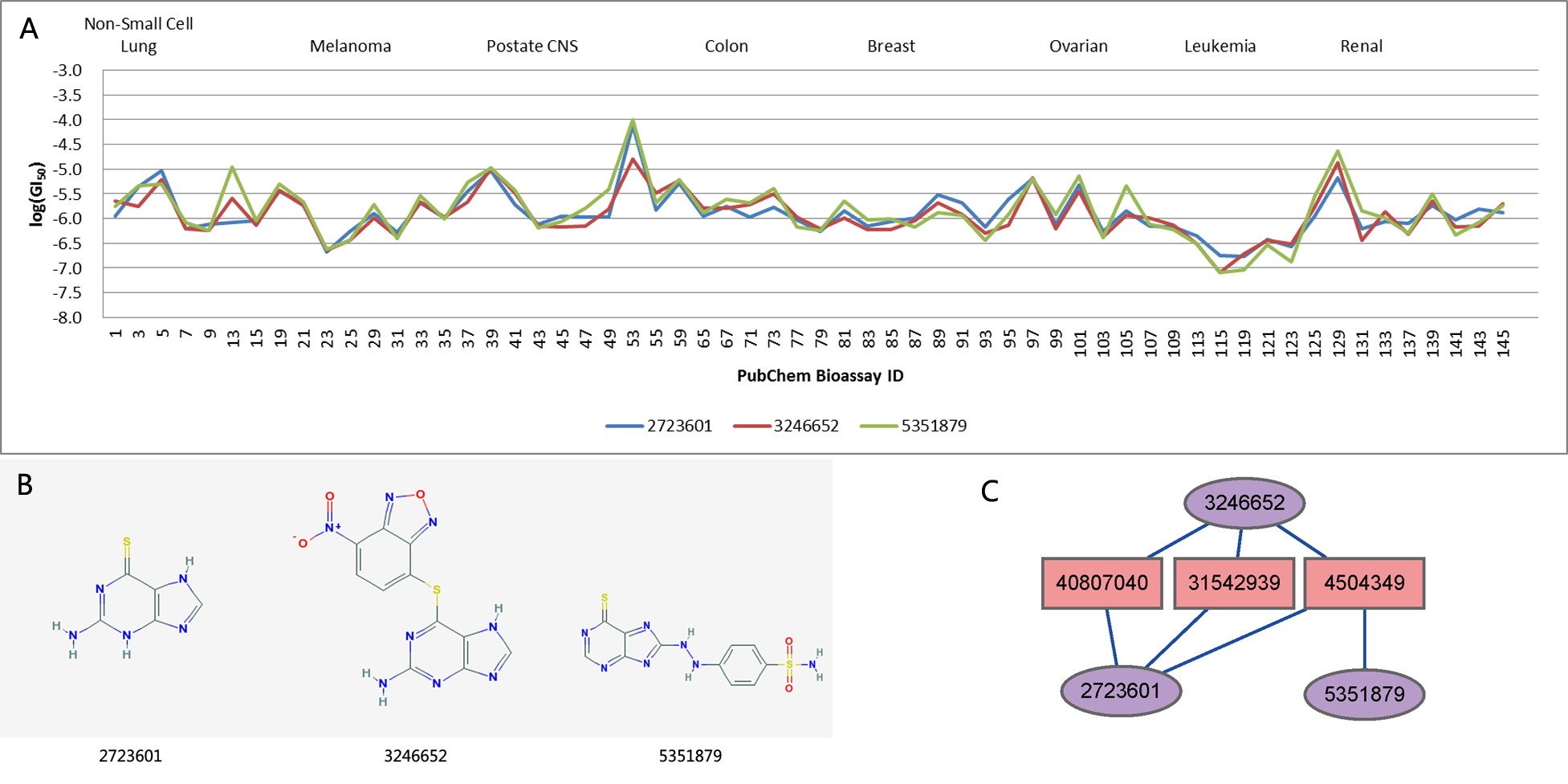


The three compounds identified from the cluster A. (A) Bioactivity profiles in the NCI-60 cell lines on nine different organs; (B) 2D chemical structures; (C) Compound-target interaction network

**Fig. S3.** Bioactivity profile, molecular structure and target information of cluster C


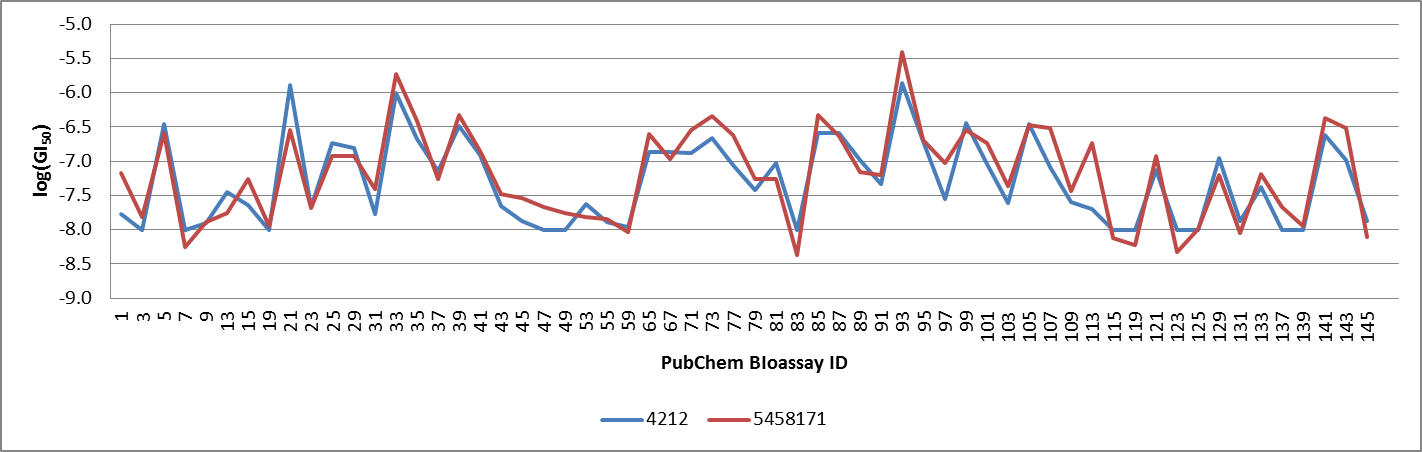

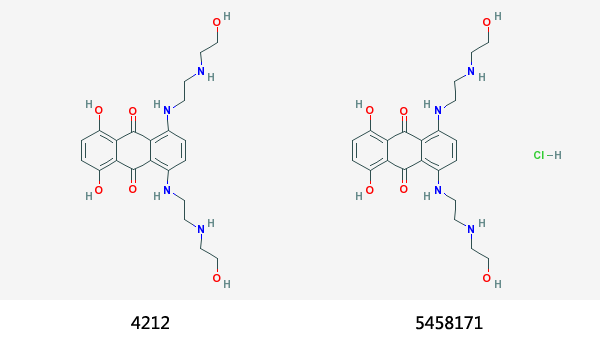

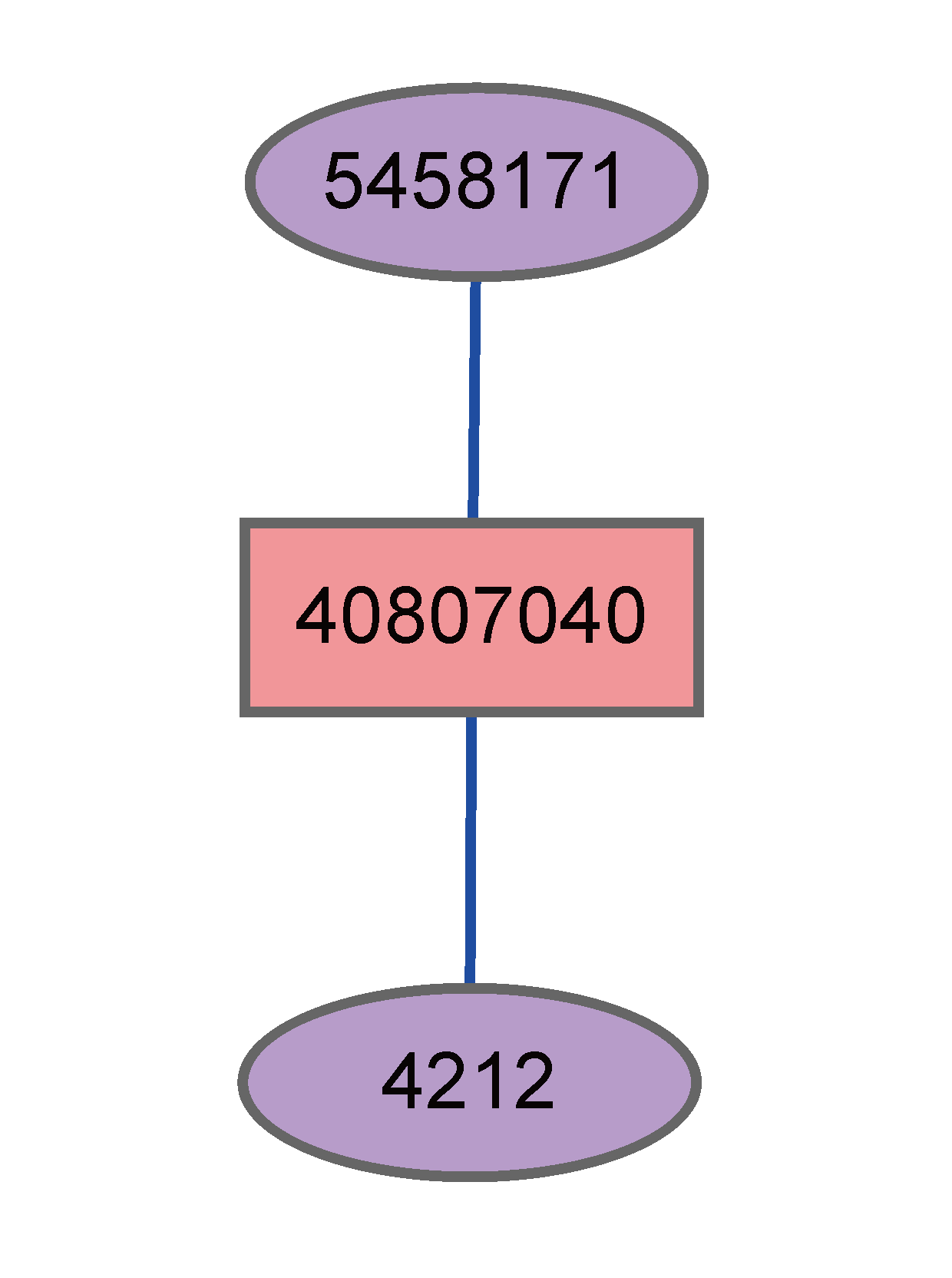


A

B

C

The three compounds identified from the cluster C. (A) Bioactivity profiles in the NCI-60 cell lines on nine different organs; (B) 2D chemical structures; (C) Compound-target interaction network
